# Long-term calorie restriction, but not endurance exercise, lowers core body temperature in humans

**DOI:** 10.18632/aging.100280

**Published:** 2011-03-27

**Authors:** Andreea Soare, Roberto Cangemi, Daniela Omodei, John O. Holloszy, Luigi Fontana

**Affiliations:** ^1^ Division of Geriatrics and Nutritional Sciences and Center for Human Nutrition, Washington University School of Medicine, St. Louis, Missouri, USA; ^2^ Department of Endocrinology and Diabetes, University Campus Bio-Medico, Rome, Italy; ^3^ Department of Experimental Medicine, University of Rome “La Sapienza,” Italy; ^4^ CEINGE Biotecnologie Avanzate, Napoli, Italy; ^5^ Division of Nutrition and Aging, Istituto Superiore di Sanità, Rome, Italy

**Keywords:** Calorie Restriction, endurance exercise, core body temperature, metabolism, thyroid hormone

## Abstract

Reduction of body temperature has been proposed to contribute to the increased lifespan in calorie restricted animals and mice overexpressing the uncoupling protein-2 in hypocretin neurons. However, nothing is known regarding the long-term effects of calorie restriction (CR) with adequate nutrition on body temperature in humans. In this study, 24-hour core body temperature was measured every minute by using ingested telemetric capsules in 24 men and women (mean age 53.7±9.4 yrs) consuming a CR diet for an average of 6 years, 24 age- and sex-matched sedentary (WD) and 24 body fat-matched exercise-trained (EX) volunteers, who were eating Western diets. The CR and EX groups were significantly leaner than the WD group. Energy intake was lower in the CR group (1769±348 kcal/d) than in the WD (2302±668 kcal/d) and EX (2798±760 kcal/d) groups (P<0.0001). Mean 24-hour, day-time and night-time core body temperatures were all significantly lower in the CR group than in the WD and EX groups (P≤0.01). Long-term CR with adequate nutrition in lean and weight-stable healthy humans is associated with a sustained reduction in core body temperature, similar to that found in CR rodents and monkeys. This adaptation is likely due to CR itself, rather than to leanness, and may be involved in slowing the rate of aging.

## INTRODUCTION

Calorie restriction (CR) without malnutrition increases lifespan and healthspan in rodents and non-human primates [1,2]. Several studies have documented a lowering of core body temperature by CR in mice, rats and rhesus monkeys [3-6]. Interestingly, ad-libitum-fed transgenic mice overexpressing the uncoupling protein 2 in hypocretin neurons (Hcrt-UCP2) also have a lower core body temperature, and a 16% greater life expectancy than wild type animals, independently of caloric intake [7]. In the Baltimore Longitudinal Study of Aging (BLSA) men with a core body temperature below the median lived significantly longer than men with body temperature above the median in the absence of CR [8].

In mammals body temperature is tightly regulated by hypothalamic neurons. The neurons located in the preoptic area integrate central and peripheral (e.g. environmental and metabolic) signals, and by modulating the autonomic and hormonal control of heat production and heat loss, maintain core body temperature nearly constant at different ambient temperatures [9]. It is well know that nutrition is a major regulator of energy production and body temperature [10]. To maintain body temperature higher than the ambient temperature, mammals utilize a substantial amount of energy. Reducing core body temperature when food availability is scarce is an effective strategy to save energy. The metabolic and molecular adaptations that mediate a reduction in core body temperature may contribute to the anti-aging effects of CR.

No information on the effects of long-term CR without malnutrition on 24-h core body temperature in humans has been published. The purpose of the present study was to measure the core body temperature in healthy, weight-stable members of the Calorie Restriction Society, who have been consuming CR diets, containing adequate protein and micronutrients, for years. Mean 24-hour, day-time and night-time core body temperatures of the CR group were compared with values obtained in two comparison groups: 1) age- and sex-matched sedentary subjects consuming a Western diet (WD), and 2) age-, sex-, and body fat-matched endurance runners consuming a WD.

## RESULTS

### Body Composition

Mean BMI values were different between the three groups (Table 1). Total body fat were similar in the CR and EX groups, and lower than in the WD group (Table 1).

**Table 1.** Characteristics of the study subjects

	CR group (n = 24)	EX group (n = 24)	WD group (n = 24)	P value
Age (yrs)	53.7±9.4	52.7±10	53.7±10.2	ns
Sex (M/F)	20/4	20/4	20/4	
Height (m)	1.74±0.1	1.75±0.1	1.79±0.1	ns
Weight (Kg)	58.2±5.9^1^^,^^2^	68.4±9.6^1^	78.7±15.5	0.0001
BMI (kg/m^2^)	19.3±1.3^1^^,^^2^	22.2±2.1^1^	24.4±2.8	0.0001
Lean mass (kg)	47.9±6.7^3^	54.5±8.9	56.5±12.9	0.010
Total body fat (%)	13.0±5.3^1^	15.2±5.1^1^	21.8±6.8	0.0001
Body surface area (m^2^)	1.70±0.11^1^^,^^4^	1.83±0.16^3^	1.97±0.25	0.0001

Values are means ± SD

1 Significantly different from Western diet group: P≤0.006

2 Significantly different from the EX group: P≤0.006

3 Significantly different from Western diet group: P≤0.03

4 Significantly different from the EX group: P≤0.05

### Nutrient intake

The CR subjects consumed a variety of foods which supplied more than 100% of the Recommended Daily Intake (RDI) for all the essential nutrients. Foods with a high nutrient-to-energy ratio such as vegetables, fruits, nuts, dairy products, egg whites, wheat and soy proteins, and lean meat were consumed, whereas processed foods, rich in refined carbohydrates, free sugars and partially hydrogenated oils, were avoided. Energy intake was lower in the CR group (1769±348 kcal/d) than in either the EX group (2798±760 kcal/d) or WD group (2302±668 kcal/d) (P≤0.0001). Energy intake in the CR group was ~23% and ~37% below that of the WD and EX groups, respectively. The percentage of total energy intake derived from protein, carbohydrate, fat and alcohol was ~21%, 50%, 29% and 0.1%, respectively in the CR group, ~17%, 49%, 32% and 2% in the EX group, and ~16%, 46%, 34% and 4% in the WD group.

### Core body temperature

**Figure 1.**
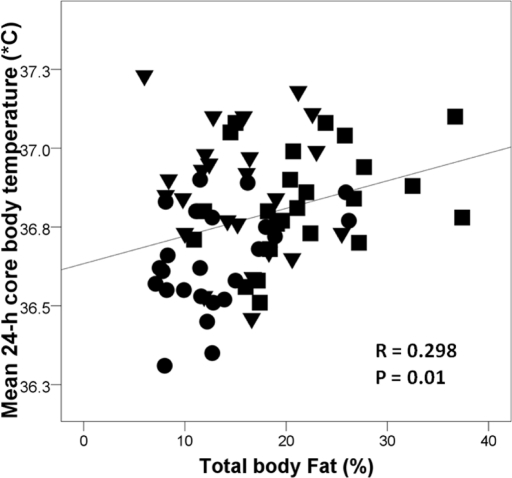
Relationship between mean 24-h core body temperature and % body fat measured by DEXA in the CR group (●), endurance runners (▼), and sedentary Western diet group (■). Pearson correlation was used to assess associations between continuous variables.

Mean 24-h, day-time and night-time core body temperature were significantly lower in the CR group than in the EX or WD groups (Table 2). Mean 24-h core body temperature correlated linearly with % body fat (r = 0.298; p = 0.01) (Figure 1), but not with age (r = 0.011; p = 0.929), body weight (r = 0.066; p = 0.581), lean body mass (r = 0.026; p = 0.834), or body surface area (r = 0.004; p = 0.976). The correlation between 24-h core body temperature and % body fat became stronger (r = 0.539; p = 0.0001) when the EX participants were excluded from the analysis. In contrast, there was no correlation between 24-h core body temperature and % body fat when the CR individuals were excluded from the analysis (r = 0.056; p = 0.706).

**Table 2.** Core body temperature

	CR group (n = 24)	EX group (n = 24)	WD group (n = 24)	P value
Mean 24-h temperature	36.64±0.16^1^^,^^2^	36.86±0.20	36.83±0.17	0.0001
Day-time temperature (8 am to 10.30 pm)	36.78±0.23^3^^,^^4^	36.97±0.22	36.95±0.26	0.01
Night-time temperature (2 to 5 am)	36.35±0.17^1^^,^^4^	36.54±0.29	36.56±0.20	0.004

Values are means ± SD

1 Significantly different from Western diet group: P≤0.006

2 Significantly different from the EX group: P≤0.0001

3 Significantly different from Western diet group: P<0.05

4 Significantly different from the EX group: P≤0.02

## DISCUSSION

In this study, mean 24-hour, day-time and night-time core body temperature were all significantly lower in the CR group than in the WD and EX groups. This reduction in 24-h core body temperature is consistent with findings in CR rodents and monkeys studies [3-6], and may contribute at least in part to the anti-aging effects of CR.

Although we found a significant correlation between % body fat and 24 h body temperature, it seems likely that the reduction in core body temperature induced by CR is largely related to CR itself, rather than changes in body composition. Mean 24-hours, day-time and night-time core body temperature were ~0.2 °C lower in the CR than in the EX group, even though percent body fat was similarly low in these groups. However, energy intake was ~37% lower in the CR than in the EX groups. It has been hypothesized that the CR-mediated reduction in body temperature relates to the induction of an energy conservation mechanism during CR [11]. In humans, monkeys and rodents long-term CR (but not endurance exercise) reduces the circulating levels of triiodothyronine [12-15] which controls energy homeostasis, body temperature, and cell respiration [16, 17]. The decrease in T3 could play a role in mediating the decrease in body temperature, and the reductions in T3 and temperature could influence the rate of aging by reducing metabolic rate and oxidative stress.

It is interesting in this context that body temperature is also reduced in the long-lived dwarf and growth hormone receptor KO mice [18, 19]. As in CR rodents, circulating levels of IGF-1, insulin and thyroid hormones are reduced in the dwarf and growth hormone receptor KO mice [20]. In contrast, in humans practicing long-term CR do not have lower circulating IGF-1 levels [21], suggesting that a down-regulation of the IGF-1 pathway is not involved in mediating the reduction in body temperature. The importance of a reduction of body temperature in modulating longevity has been also supported by the data obtained from the Hcrt-UCP2 mice and the Baltimore Longitudinal Study of Aging (BLSA). Overexpression of the uncoupling protein 2 in hypocretin neurons causes an elevation of hypothalamic temperature that leads to a 0.3-0.5°C reduction in core body temperature, and a significant increase in longevity, independently of caloric intake [7]. In the Baltimore Longitudinal Study of Aging (BLSA), men with core body temperatures below the median lived significantly longer than men with body temperatures above the median in the absence of CR [8].

Data from weight loss studies have shown that short-term (6 mos) CR significantly decreases core body temperature in overweight subjects that are actively losing weight [22]. However, in overweight men and women who had achieved a “stable” lower body weight using a low-calorie liquid diet, there was no change in core body temperature [22]. Consistently, no difference in core body temperature between weight-stable obese and normal-weight subjects has been detected, suggesting that in steady-state obese subjects maintain their core body temperature normal probably by increasing heat dissipation from peripheral regions [23, 24]. In contrast, in our study we have found that long-term CR, but not endurance exercise, chronically reduces core body temperature in weight-stable lean individuals. One possible explanation for the reduced 24-h core body temperature in the CR practitioners may be a protective physiological adaptation to save energy. This hypothesis is supported by the finding that the reduced core body temperature in the CR group is associated with lower circulating levels triiodothyro-nine, insulin, leptin and total testosterone, which are key nutrient-sensing metabolic/anabolic hormones [12, 25, 26]. The combination of decreased core body temperature and lower circulating levels of serum triiodothyronine, leptin, and anabolic hormones (i.e. testosterone, insulin) is a clear indication that these individuals are in a state of “sensing” severe energy restriction.

In conclusion, the results of this study provide evidence that long-term CR, with adequate intake micronutrients, in healthy, weight-stable individuals is associated with a reduction of mean 24-hour, day-time and night-time core body temperature, similar to that found in calorie-restricted rodents and monkeys.

## METHODS

### Study participants

Three groups (24 participants/group) were studied. One group (CR group) had been consuming a CR diet with adequate nutrients for a 6±3 years (range 3-15 yrs) and were members of the Calorie Restriction Society, who we asked to come to St.Louis to participate in this study. The CR Society members practice severe CR, because they believe that CR will markedly increase their disease-free longevity. Most of them live in North America, although there are also some CR Society members in England, Scandinavia and Japan. The second group (EX group), were endurance runners who had been running an average of 46 miles/wk (range 22-89 miles/wk) for 20±10 yrs (range 7-34 yrs), and were recruited from the St.Louis area. The EX group was matched on age, sex, and percent body fat with the CR group. The third group (WD group) were healthy sedentary (regular exercise <1h/week) normal weight individuals, recruited from the St.Louis area, who were eating a WD. The WD group was matched on age and sex with the CR and EX groups. The characteristics of the study participants are shown in Table 1. None of the participants had evidence of chronic disease, smoked cigarettes, or were taking medications that could affect the outcome variables. All participants reported weight stability, defined as less than a 2-kg change in body weight in the preceding 6-mos. This study was approved by the Human Studies Committee of Washington University School of Medicine, and all participants gave informed consent before their participation.

### Body composition

Total body fat mass and fat free mass were determined by dual-energy X-ray absorptiometry (DXA) (QDR 1000/w, Hologic, Waltham, MA).

### Dietary assessment

Participants recorded all food and beverage intakes for 7 consecutive days. Food records were analysed by using the NDS-R program (version 4.03_31).

### Core body temperature measurement

Twenty-four hour core body temperature was measured every minute using radiofrequency telemetered thermometers (CorTemp, HQ Inc, Palmetto, FL). Each thermometer pill contains a crystal quartz oscillator which transmits core temperature readings within ± 0.1 °C through a low frequency radio wave to an external receiver/data logger [27]. All the subjects tested were on a stable diet, at a stable weight, and without acute illness or recent hospitalization. The participants were instructed to refrain from exercise for at least 48 hours prior to CorTemp pill ingestion. Mean 24-hour, daytime (8 AM-10:30 PM), and nighttime (2 AM-5 AM) temperatures were computed.

### Statistical analysis

One-way analysis of variance (ANOVA) was used to compare group variables, followed by Tukey post-hoc testing when indicated. One-way ANOVA with Games-Howell was performed for distributions where equal variances could not be assumed. Statistical significance was set at P < 0.05 for all tests. All data were analyzed by using SPSS software, version 13.0 (SPSS Inc, Chicago). All values are expressed as means±SD.
